# Household Transmission of SARS-CoV-2

**DOI:** 10.1001/jamanetworkopen.2021.0304

**Published:** 2021-02-26

**Authors:** Joshua P. Metlay, Jennifer S. Haas, Alexander E. Soltoff, Katrina A. Armstrong

**Affiliations:** 1 Division of General Internal Medicine, Department of Medicine, Massachusetts General Hospital, Harvard Medical School, Boston, Massachusetts

## Abstract

This cohort study assesses the risk of household transmission of SARS-CoV-2 and the associated risk factors among exposed household members.

## Introduction

The primary mode of spread of severe acute respiratory syndrome coronavirus 2 (SARS-CoV-2)—the virus that causes coronavirus disease 2019 (COVID-19)—is by person-to-person contact via respiratory droplets.^1^ Given that most virus transmission has occurred in homes,^2^ we investigated whether home addresses recorded in the electronic medical record could be used to accurately estimate transmission risk and identify risk factors for transmission.

## Methods

We conducted a retrospective cohort study of COVID-19 risk among exposed children and adults in households where an index case of COVID-19 was diagnosed between March 4 and May 17, 2020. The study was conducted within the Mass General Brigham system, a large integrated hospital and ambulatory care network based in Boston, Massachusetts. No participant contact was required for this study, which was determined to be exempt and for which patient informed consent was waived by the Mass General Brigham institutional review board. This study followed the Strengthening the Reporting of Observational Studies in Epidemiology (STROBE) reporting guideline.

Index cases were identified on the basis of a positive reverse transcriptase–polymerase chain reaction test result for SARS-CoV-2. Patients whose home addresses could not be geocoded were excluded. The at-risk cohort was compiled on the basis of identifying all patients registered at the addresses of index cases (including unit numbers) and excluded all patients who did not have at least 1 visit to the health system within the preceding 60 months. Addresses with more than 25 identified residents were excluded, although we included sensitivity analyses with smaller household sizes to test the validity of the home address field. Follow-up for all eligible patients continued until the end of the study period or until they had a positive SARS-CoV-2 test result documented in the system.

Characteristics of household contacts were extracted from the electronic medical record. The comparison of risk between groups was conducted with either the χ^2^ or Mantel-Haenszel χ^2^ test for trend as appropriate. Mixed-effects logistic regression models were used to calculate the adjusted infection risk, accounting for clustering of exposed subjects within households. Two-sided *P* < .05 was considered to be statistically significant. Analyses were performed using Stata SE version 16.1 (StataCorp LLC).

## Results

The 7262 index cases were linked to 17 917 additional at-risk individuals assigned to the same addresses. These at-risk individuals consisted of 9341 (52.1%) females and 8576 (47.9%) males; 6888 (38.4%) resided in households of 6 to 10 people. Their mean (SD) age was 36.1 (23.3) years, and 4672 (26.1%) were 18 years or younger (Table 1). Within this exposed cohort, 1809 cases of COVID-19 were subsequently diagnosed, resulting in an overall incidence of COVID-19 of 10.1% (Table 1). The median time to diagnosis from the date of the index case was 3 days (interquartile range, 1-9 days).

**Table 1.** Coronavirus Disease 2019 Infection Risk Among 17 917 Exposed Household Members

Characteristic	Individuals exposed, No.	Individuals with infection, No. (%)^a^	*P* value^b^
Age group, y			
≤18	4672	210 (4.5)	<.001
19-29	3471	302 (8.7)	
30-49	4322	547 (12.7)	
50-64	3160	464 (14.7)	
65-74	1134	154 (13.6)	
≥75	1158	132 (11.4)	
Sex			
Female	9341	986 (10.6)	.03
Male	8576	823 (9.6)	
Household size, No. of members			
2	1339	185 (13.8)	<.001
3-5	7061	763 (10.8)	
6-10	6888	662 (9.6)	
11-19	2358	184 (7.8)	
20-25	271	15 (5.5)	
Comorbid conditions			
Asthma	2280	323 (14.2)	<.001
Cancer	348	73 (21.0)	<.001
Cardiovascular disease	437	91 (20.8)	<.001
Chronic obstructive pulmonary disease	798	116 (14.5)	<.001
Dementia	85	15 (17.7)	.02
Diabetes	1440	288 (20.0)	<.001
HIV	71	9 (12.7)	.90
Hypertension	1271	274 (21.6)	<.001
Liver disease	235	60 (25.5)	<.001
Obesity	3727	543 (14.6)	<.001
Kidney disease	225	54 (24.0)	<.001

^a^ Percentages indicates the row percentage. A total of 1809 exposed individuals were infected.

^b^ *P* values were calculated with either the χ^2^ or Mantel-Haenszel χ^2^ test for trend.

Independent factors significantly associated with higher transmission risk included age greater than 18 years (eg, adjusted odds ratio [OR] for those aged 50-64 years, 3.66; 95% CI, 2.92-3.66; *P* < .001) and multiple comorbid conditions (eg, adjusted OR for individuals with hypertension, 1.93; 95% CI, 1.58-2.44; *P* < .001) (Table 2). In sensitivity analyses limiting the maximum size of the household to as small as 2 persons, the calculated transmission risk increased to only 13.8%.

**Table 2.** Independent Risk Factors for COVID-19 Among Exposed Household Members

Characteristic	Adjusted OR (95% CI)^a^	*P* value
Age group, y		
≤18	1 [Reference]	
19-29	2.13 (1.70-2.66)	<.001
30-49	3.48 (2.83-4.26)	<.001
50-64	3.66 (2.92-4.59)	<.001
65-74	2.73 (2.03-3.66)	<.001
≥75	1.85 (1.34-2.55)	<.001
Sex		
Female	1 [Reference]	
Male	0.89 (0.79-1.01)	.08
Household size, members		
2	1 [Reference]	
3-5	0.80 (0.63-1.02)	.07
6-10	0.76 (0.59-0.97)	.03
11-19	0.59 (0.42-0.82)	.002
20-25	0.32 (0.12-0.81)	.02
Comorbid conditions		
Asthma	1.31 (1.07-1.59)	.008
Cancer	1.61 (1.12-2.33)	.01
Cardiovascular disease	1.45 (1.02-2.06)	.04
Diabetes	1.67 (1.36-2.06)	<.001
Hypertension	1.93 (1.58-2.44)	<.001
Liver disease	2.01 (1.32-3.07)	.001
Obesity	1.35 (1.15-1.57)	<.001

Abbreviation: OR, odds ratio.

^a^ Adjusted for other comorbid conditions, including chronic obstructive pulmonary disease, HIV, kidney disease, and dementia.

## Discussion

Our study showed an overall household infection risk of 10.1%, consistent with reported transmission risk based on more traditional contact tracing, including a recent meta-analysis that reported an overall transmission risk of 17.1%, although there was wide variation across studies.^3,4^

The major limitation of our study is that we relied on home addresses within our electronic medical records, which likely led to both undercounting and overcounting of household members. Although we acknowledge that contact investigations are the standard approach for estimating household transmission risk, we believe that the consistency of our results with these approaches suggests that our approach may provide a more efficient method for risk estimation and household contact identification. Moreover, our sensitivity analysis indicated that the results were qualitatively similar when restricted to smaller households. In addition, because testing of household members was driven by care-seeking behavior, it is likely that we did not observe all infections that occurred in the households. In addition, the relatively short median interval between the index case and infected household members suggests that some household infections were due to common exposures rather than transmission from one household member to another.

SARS-CoV-2 transmission in US households is a major source of new infections. Electronic medical record data may support intensive infection control efforts.
